# Helminth infection modulates the immunogenicity of COVID-19 vaccines in mice without compromising protective efficacy

**DOI:** 10.3389/fimmu.2026.1827532

**Published:** 2026-05-28

**Authors:** Jinpeng Su, Youssef Hamway, Daniele Mistretta, Bo-Hung Liao, Zhe Ma, Julia Schluckebier, Zhe Xie, Olga Polezhaeva, Walaa Jradi, Sarah Braun, Jennifer Robb, Laura Franziska Main, Piyas Mukherjee, Lorna Jubran-Rudolf, Katja Steiger, Paco Pino, Matthias Tenbusch, Elvira D’Ippolito, Gregor Ebert, Ulrike Protzer, Clarissa Prazeres da Costa

**Affiliations:** 1 Institute of Virology, School of Medicine and Health, Technical University of Munich/Helmholtz Munich, Munich, Germany; 2 School of Medicine and Health, German Center for Infection Research (DZIF), Munich Partner Site, Munich, Germany; 3 Institute for Medical Microbiology, Immunology and Hygiene, School of Medicine and Health, Technical University of Munich, Munich, Germany; 4 Center for Global Health, TUM School of Medicine and Health, Technical University of Munich, Munich, Germany; 5 Comparative Experimental Pathology, School of Medicine and Health, Technical University of Munich, Munich, Germany; 6 Institute of Pathology, School of Medicine and Health, Technical University of Munich, Munich, Germany; 7 School of Medicine and Health, ExcellGene SA, Monthey, Switzerland; 8 Harald zur Hausen Institute of Virology, Uniklinikum Erlangen, Friedrich-Alexander-Universität Erlangen-Nürnberg (FAU), Erlangen, Germany; 9 FAU Profile Center Immunomedicine, Friedrich-Alexander-Universität Erlangen-Nürnberg, Erlangen, Germany

**Keywords:** COVID-19, helminth infection, immunogenicity, mRNA vaccine, protective efficacy, protein vaccine, SARS-CoV-2, *Schistosoma mansoni* infection

## Abstract

**Objectives:**

Helminth parasites infect over a quarter of the global population and can profoundly modulate host immunity, potentially influencing vaccine performance and the spread of pandemic pathogens such as severe acute respiratory syndrome coronavirus 2 (SARS-CoV-2). Despite the high global endemicity of helminth infections, their impact on immune responses to various COVID-19 vaccines remains unknown. This study aimed to evaluate the impact of *Schistosoma* infection on the immunogenicity and protective efficacy of messenger RNA (mRNA)- and protein-based COVID-19 vaccines.

**Methods:**

Mice with *Schistosoma* infection and non-infected controls were immunized with either an mRNA-based COVID-19 vaccine or an alum-adjuvanted spike protein vaccine. Vaccine-induced humoral and cellular immune responses were assessed, and protective efficacy was evaluated using a SARS-CoV-2 challenge model.

**Results:**

COVID-19 mRNA vaccination induced strong spike-specific antibody and CD4 T-cell responses in *Schistosoma*-infected mice comparable to non-infected controls, despite a Th2/regulatory-biased immune environment, although multifunctional CD8 T-cell responses were reduced. Alum-adjuvanted protein vaccination elicited robust humoral but weaker cellular immunity, with comparable immune responses in infected and non-infected mice. Following SARS-CoV-2 challenge, both vaccine platforms conferred effective protection, with substantial viral clearance and minimal lung pathology.

**Conclusions:**

mRNA and protein vaccines elicit distinct immune profiles; however, both protect effectively against SARS-CoV-2 infection in mice with concurrent helminth infection.

## 1 Introduction

The rapid development of vaccines across multiple technological platforms defined the global response to COVID-19. Of these, messenger RNA (mRNA) and viral vector vaccines elicit strong neutralizing antibody responses, along with robust cellular immunity (1), including cytotoxic CD8 T cells and Th1-polarized CD4 T-cell responses (2–4). In contrast, protein-subunit and inactivated virus vaccines rely predominantly on humoral immunity (5–7). In clinical Phase III trials and real-world studies, these platforms demonstrated efficacies often exceeding 85% for both protein-based (5) and mRNA vaccines (8, 9).

However, most clinical trials were conducted outside of settings where chronic parasitic infections are endemic. Although low- and middle-income countries account for approximately 85% of the global population, fewer than one-fifth of severe acute respiratory syndrome coronavirus 2 (SARS-CoV-2) vaccine trials have been conducted in these regions (10). This leaves significant gaps in understanding how host or environmental factors common to these populations influence vaccine performance (11). Helminth infections are among the most prevalent chronic infections worldwide, affecting an estimated 3.5 billion people, and are well known to modulate immune and vaccine responses (12). Schistosomiasis alone affects more than 250 million individuals (13), primarily in sub-Saharan Africa, where *Schistosoma mansoni* and *Schistosoma haematobium* establish chronic, immune-modulating infections.

The immunological landscape of *Schistosoma* infection evolves in tandem with the parasite development in the host. The early phase, marked by larval migration, elicits innate and Th1-associated inflammation. With the onset of egg deposition (6–8 weeks post-infection), the immune response shifts toward a type 2 profile characterized by interleukin-4 (IL-4), IL-5, and IL-13 production; eosinophil recruitment; and granuloma formation around tissue-trapped eggs. As the infection becomes chronic after approximately 12 weeks, a subsequent transition toward a regulatory immune environment occurs, dominated by IL-10 and transforming growth factor-β (TGF-β), primarily driven by the expansion of regulatory T and B cells, and tolerogenic antigen-presenting cells (14). This milieu dampens antigen presentation and suppresses the Th1/follicular T helper (Tfh) and cytotoxic responses required for many viral vaccines (15).

The possibility that chronic helminth infection alters SARS-CoV-2 vaccine immunogenicity was raised during the pandemic (16); yet, to date, there are no human trials and very few experimental studies addressing this question (17). Human and animal studies have shown that helminth infections can attenuate responses to vaccines such as Bacillus Calmette-Guérin (BCG), tetanus toxoid, influenza, and hepatitis B, often through IL-10- and TGF-β-mediated suppression of Th1 and Tfh responses. In some instances, these effects are partially reversible following anthelmintic treatment (12). However, data on respiratory viruses remain sparse. In the murine helminth infection model of *Litomosoides sigmodontis*, seasonal influenza virus vaccination resulted in reduced antibody quantity and quality, driven by IL-10-producing regulatory CD4 T cells (18). Similarly, murine *Heligmosomoides polygyrus* infection dampened polyfunctional CD4 and CD8 T-cell responses to mRNA SARS-CoV-2 vaccination and compromised protection after viral challenge (17). These findings support the hypothesis that chronic helminth infection could selectively impair the cellular and qualitative components of antiviral vaccine immunity. However, some critical questions remain: (1) whether other types of commonly used vaccines with differing immunogenicity, such as protein-subunit vaccines, would be affected by underlying helminth infections, and (2) whether other types of helminths would equally influence vaccine immunogenicity. To our knowledge, no studies have examined SARS-CoV-2 vaccines in the context of a human-relevant helminth, despite the high prevalence of schistosomiasis.

Here, we address these gaps using a murine model of *S. mansoni* infection. Specifically, we investigated how chronic helminth infection influences both the immunogenicity and protective efficacy of COVID-19 vaccination across distinct vaccine platforms. We compared the immunogenicity and protective efficacy of an mRNA vaccine (BNT162b2/Comirnaty) and of an alum-adjuvanted spike-trimer protein vaccine against challenge with the mouse-adapted SARS-CoV-2 MA20 strain (19), assessing humoral, cellular, and innate responses during different stages of *Schistosoma* infection. By directly evaluating vaccine platform-specific immune responses and protection in the context of helminth-induced immune modulation, this study provides mechanistic insight and a translational framework for optimizing vaccination strategies in parasite-endemic settings.

## 2 Materials and methods

### 2.1 Ethical statement

Animal experiments were conducted in strict accordance with the German regulations of the Society for Laboratory Animal Science (GV-SOLAS) and the European Health Law of the Federation of Laboratory Animal Science Associations (FELASA). Experiments were approved by the District Government of Upper Bavaria (permission numbers: ROB-55.2-2532.Vet_02-20-193, ROB-55.2-2532.Vet_02-21-169, ROB-55.2-2532.Vet_03-22-19, and ROB-55.2-2532.Vet_02-22-139). Mice were kept in biosafety level 1 or 3, specific pathogen-free animal facilities following institutional guidelines.

### 2.2 Animals, *Schistosoma* infection, and determination of *Schistosoma* egg burden

Eight- to 10-week-old female C57BL/6J mice were purchased from Inotiv Inc. (Indiana, USA). C57BL/6J mice were infected with a Brazilian strain of *Sm* with 80 cercariae/mouse (snail strain-*Biomphalaria glabrata*) as previously described (20–22). Infection was assessed by egg burden as described previously (20). Paraffin-embedded sections from the left liver lobe of each mouse were stained with hematoxylin and eosin (H&E) for histopathological evaluation of tissue damage.

### 2.3 Preparation of vaccine formulations and mouse immunization protocols

The mRNA-based Comirnaty (Pfizer-BioNTech) vaccine, which encodes the wild-type SARS-CoV-2 spike protein, was prepared by thawing the vial from storage at −80 °C to room temperature (RT), gently inverting it 10 times to ensure homogeneity, and then diluting it with 1.8 mL of sterile 0.9% sodium chloride solution. Afterwards, the diluted vaccine was mixed gently by inversion and aliquoted for further dilution to the appropriate doses for administration in mice.

The recombinant, trimeric spike protein is based on Prolin-based stabilizing mutations (2 Pro, 6 Pro), which keep the spike in the “closed” pre-fusion conformation, i.e., the most immunogenic form prior to fusion with the target cell receptors. The constructs carry a deletion of the transmembrane and intracellular section of the molecule, which is replaced with a short trimerization domain. Proteins were produced in stable, transfected CHO cells (CHOExpress^®^ cells, a proprietary ExcellGene host system) that secreted the constructs as fully assembled trimers into the supernatant of the production cell culture. Trimers were purified and concentrated via an affinity column that recognized and bound the molecule via the RBD region, and stored at −80°C.

The alum-adjuvanted trimeric spike protein was formulated as described previously (23). Briefly, the alum-adjuvanted spike protein vaccine was prepared by mixing 25 μL of 10 mg/mL aluminum hydroxide (Alhydrogel 2% gel, InvivoGen, USA) with 25 μL of saline solution, followed by vortexing at high speed for 5 s to obtain the alum adjuvant. Subsequently, 10 μg of spike protein in 50 μL of saline solution was combined with 50 μL of the prepared alum adjuvant and vortexed at high speed for 5 s to ensure uniform mixing prior to use.

*S*. *mansoni* (*Sm*)*-*infected and non-infected C57BL/6J mice were immunized intramuscularly at weeks 0 and 4 with 1 or 5 μg of mRNA-based Comirnaty vaccine, or 10 μg of alum-adjuvanted spike protein administered immediately after formulation without further storage. *Sm*-infected and non-infected mice without vaccination served as controls. After 1 or 3.5 weeks of the boost immunization, the mice were sacrificed for the final analyses of vaccine-induced immune responses.

### 2.4 Analysis of serum spike-specific IgG titers and subclasses by ELISA

Spike-specific IgG concentrations in mouse sera collected at the experimental endpoint were quantified using a previously described enzyme-linked immunosorbent assay (ELISA) method (24). Briefly, ELISA plates were coated overnight at 4 °C with 100 μL of 500 ng/mL spike protein (SinoBiological, China), washed with phosphate-buffered saline (PBS) containing 0.05% Tween 20 (PBST), and blocked with 200 μL of 5% fetal calf serum (FCS) in PBS for 2 h at RT. Diluted mouse sera with optimal dilution factors were added to the wells and incubated for 2 h at RT. After washing, wells were incubated for 1 h at RT with 100 μL of horseradish peroxidase (HRP)-conjugated antibodies: goat anti-mouse IgG (Sigma-Aldrich, 1:2,000 in PBS) for total IgG, or goat anti-mouse IgG1 and IgG2c (SouthernBiotech, 1:1,000 in PBS) for subclass analysis. For the standard curve of IgG, plates were coated with serial dilutions of mouse IgG (Sigma-Aldrich, Germany), starting at 500 ng/mL, blocked, incubated with PBS for 2 h, and then treated with HRP-conjugated goat anti-mouse IgG. After five washes, 100 μL of stabilized TMB chromogen solution was added to each well. Plates were incubated in the dark for 2–3 min, and the reaction was stopped by adding 100 μL of 2 N sulfuric acid per well. Optical density was then measured at 450 nm with background subtraction at 560 nm using a plate reader (Tecan Infinite F200, Tecan, Germany).

### 2.5 Intracellular cytokine staining of murine splenocytes

Murine splenocytes were isolated by mechanical dissociation of spleens through 100-µm cell strainers, followed by erythrocyte lysis using ammonium–chloride–potassium (ACK) buffer for 1 min at RT.

For intracellular cytokine analysis, up to 2 × 10^6^ splenocytes were stimulated overnight with 1 µg/mL PepMix™ SARS-CoV-2 Spool1 or Spool2 peptide pools (JPT, Germany) in the presence of brefeldin A (BFA). Cells stimulated with the ovalbumin-derived peptide SIINFEKL (OVAS8L) served as negative controls. The following day, cell-surface staining was performed using anti-CD4 and anti-CD8 antibodies. Dead cells were excluded by staining with Fixable Viability Dye eF780 (eBioscience, Germany). Afterwards, cells were fixed and permeabilized, and intracellular staining for interferon γ (IFNγ), tumor necrosis factor-α (TNF-α), and IL-2 was performed. Data were acquired on a CytoFLEX S flow cytometer (Beckman Coulter, USA) and analyzed using FlowJo software (Tree Star, USA).

### 2.6 Detection of spike- or *Schistosoma*-specific Th1/Th2 cytokine profiles in murine splenocyte supernatants

Up to 2 × 10^6^ freshly isolated murine splenocytes per well were seeded into flat-bottom 96-well plates. For the stimulation, 2 μg/mL of spike protein or 20 µg/mL of *Schistosoma* egg antigen (SEA) were added to the cells in a final volume of 300 μL per well, as described previously (22). After 48-h incubation at 37 °C, the supernatants were harvested to determine the concentration of secreted spike-specific Th1 (IFNγ, TNFα^+^, and IL-2) and Th2 cytokines (IL-4 and IL-5) using the multiplex assay of LEGENDplex™ MU Th Cytokine panel (BioLegend, USA), according to the manufacturer’s instructions, or SEA-specific IFNγ, IL-5, and IL-10 using ELISA (Thermo Fisher 88-7314, 88-7054, and 88-7105), according to the manufacturer’s instructions.

### 2.7 Macrophage analyses in lung tissue

Lung tissue was dissociated, and cell suspensions were prepared using the Lung Dissociation Kit and gentleMACS system (Miltenyi Biotec, Germany), according to the manufacturer’s instructions. After counting, cells were stained with a panel of myeloid antibodies (CD64, CD11c, CD11b, F4/80, MHC-II, XCR1, Sirpα, CD80, and CD86) and acquired on a Cytoflex LX (Beckman Coulter).

### 2.8 Serum SARS-CoV-2 neutralization activity

Serum neutralization activity was assessed using a cell culture-based infection inhibition assay, as previously described (25). Briefly, high-titer SARS-CoV-2 Munich-TUM-1 virus stocks were generated by infecting Vero E6 cells (ATCC, USA) maintained in Dulbecco’s Modified Eagle Medium (DMEM) supplemented with 10% FCS, 1% penicillin/streptomycin, 200 mmol/L L-glutamine, 1% MEM non-essential amino acids, and 1% sodium pyruvate (all from Gibco, Germany). For the neutralization assay, Vero E6 cells were seeded at 15,000 cells per well in 96-well plates and incubated overnight at 37 °C with 5% CO_2_. On the day of infection, serum samples were serially diluted 1:2 in fresh medium (25 µL) and mixed with an equal volume (25 µL) of SARS-CoV-2 virus at a multiplicity of infection (MOI) of 0.03. The virus–serum mixtures were pre-incubated for 1 h at 37 °C before being transferred to the pre-seeded Vero E6 cells for an additional 1-h incubation. Subsequently, the inoculum was removed, and cells were cultured in supplemented DMEM for 23 h at 37 °C. After incubation, infections were terminated by fixing cells with 4% paraformaldehyde, and viral infection rates were quantified by in-cell ELISA using the SARS-CoV-2-N-specific T62 antibody (Sinobiological, China) as previously described (25). Neutralization curves were generated in GraphPad Prism (GraphPad Software Inc., USA), and 50% inhibitory concentrations (IC_50_) were determined by nonlinear regression analysis.

### 2.9 SARS-CoV-2 challenge of mice

After the completion of vaccination, all the *Sm*-infected and non-infected mice were transferred to a BSL-3 facility for SARS-CoV-2 virus challenge. The mice were aerosol-inoculated with 5 × 10^5^ PFU mouse-adapted SARS-CoV-2 MA20 strain in a total volume of 6 mL. Mice infected with SARS-CoV-2 were observed daily for clinical symptoms and body weight loss. Mice were euthanized at the indicated time points by isoflurane overdose.

### 2.10 Immunohistochemistry of SARS-CoV-2-infected mice

Lung tissues were fixed for 48 h in 10% neutral-buffered formalin, processed on an automated tissue processor (ASP300S, Leica), embedded in paraffin, and sectioned at 2 µm on a rotary microtome (HM355S, Thermo Fisher). For H&E staining, sections were deparaffinized, rehydrated through graded alcohols, stained with Mayer’s H&E using standard protocols, dehydrated, cleared in xylene, and mounted with Pertex.

Immunohistochemistry was performed on a Bond RXm autostainer (Leica) with heat-induced epitope retrieval in citrate buffer (Epitope Retrieval Solution 1) and peroxidase block. Sections were incubated with anti-SARS-CoV-2 N protein (T62, SinoBiological, 1:10,000, 15 min), and detection was performed using the Bond Polymer Refine system and DAB chromogen, followed by hematoxylin counterstaining, dehydration, and xylene mounting.

Whole-slide images were acquired with a Leica Aperio AT2 scanner and analyzed using Aperio ImageScope. Quantification of positive staining was performed in QuPath (v0.6.0) with automated tissue segmentation and a single-threshold classifier to determine the percentage of positive cells (26).

### 2.11 SARS-CoV-2 RNA extraction and quantitative real-time PCR

SARS-CoV-2 RNA in lung tissue of SARS-CoV-2-infected mice was quantified as described previously (27). Briefly, up to 30 mg of lung tissue was placed in 2-mL reinforced screw-cap tubes containing 2.8-mm ceramic beads (VWR, USA) and 500 µL of RNAlater buffer (Qiagen, Germany), and stored at −80 °C until processing. Total RNA was extracted using the NucleoSpin RNA kit (Macherey-Nagel, Germany) following the manufacturer’s protocol. RNA concentration and purity were determined with a NanoDrop spectrophotometer (Thermo Fisher, USA), and complementary DNA (cDNA) was synthesized using the PrimeScript RT Master Mix (Takara Bio, USA).

Absolute quantification of SARS-CoV-2 RNA was performed by real-time PCR with PowerTrack SYBR Green Master Mix on a QuantStudio 5 system (Thermo Fisher, USA), using a plasmid standard containing the SARS-CoV-2 N gene at serial dilutions ranging from 10^9^ to 10^1^ copies. Reactions were run with 1 mM forward (5′-GACCCCAAAATCAGCGAAAT-3′) and reverse (5′-TCTGGTTACTGCCAGTTGAATCTG-3′) primers targeting CDC-N1 (2019-nCoV_N1) under the following cycling conditions: 95 °C for 15 s, 55 °C for 10 s, and 72 °C for 25 s, repeated for 45 cycles. Data were analyzed using the QuantStudio Design and Analysis (DA1) software.

### 2.12 Statistical analyses

In all graphs, data are presented as mean ± SEM. Statistical analyses were performed using GraphPad Prism version 10 (GraphPad Software Inc., San Diego, CA). *p*-values <0.05 were considered significant. Details of biological replicates and statistical analyses are provided in the corresponding figure legends.

## 3 Results

### 3.1 *Schistosoma* infection alters immune responses to COVID-19 mRNA vaccination

**Figure 1:**
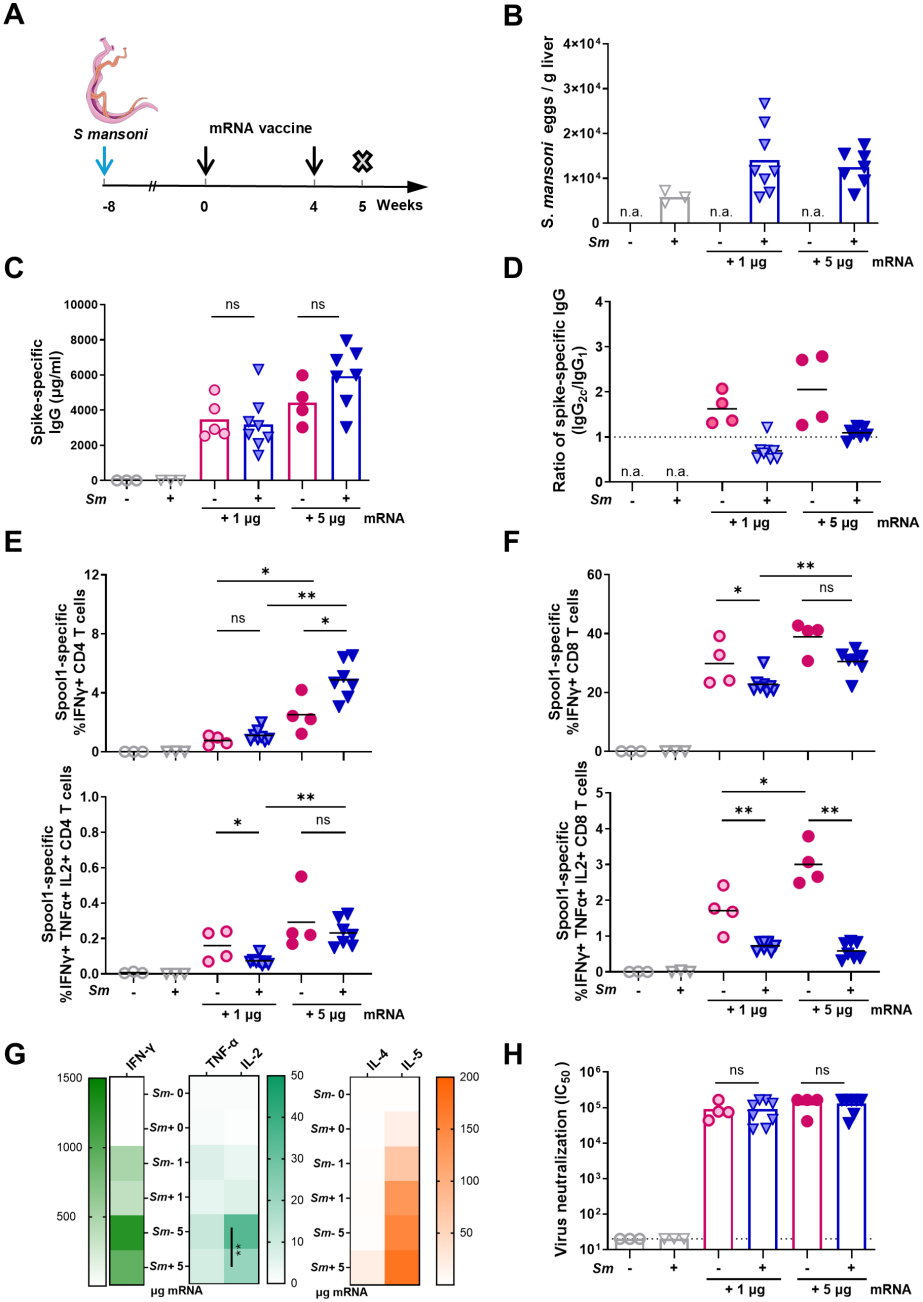
Immunogenicity of mRNA vaccine in mice with 8-week *Schistosoma* infection. **(A)** Schematic depiction of *Schistosoma* infection and vaccination regimen. C57BL/6 mice were infected with 80 *S. mansoni* (*Sm*) cercariae subcutaneously 8 weeks before the first immunization. At weeks 0 and 4, *Sm*-infected and non-infected mice (*n* ≥ 4) were immunized intramuscularly with 1 µg or 5 µg of mRNA-based Comirnaty vaccine. *Sm-*infected and non-infected mice (*n* = 3) without vaccination served as controls. One week after boost immunization (week 5), mice were sacrificed to evaluate vaccine-induced immune responses. **(B)** The *Sm* egg counts per gram liver of *Sm*-infected mice. **(C)** Amounts and **(D)** IgG_2c_/IgG_1_ ratio of SARS-CoV-2 spike-specific IgG antibodies in the sera from vaccinated mice at week 5. **(E, F)** Percentages of spike-specific IFNγ^+^ and IFNγ^+^TNFα^+^IL-2^+^ CD4 **(E)** and CD8 **(F)** T-cell responses determined by intracellular cytokine staining (ICS) of murine splenocytes following stimulation with a spike-specific peptide pool at week 5. **(G)** Cytokine levels secreted into the supernatants of spike protein stimulated splenocytes within 48 h. Data are presented as fold change compared to the non-infected group. Significant differences between paired *Sm−* and *Sm+* groups are indicated. **(H)** SARS-CoV-2 virus infection neutralization capacity of the murine sera from the vaccinated mice at week 5. Data are presented as mean ± SEM. Statistical analyses utilized the one-way ANOVA (for egg burden), Mann–Whitney, Kruskal–Wallis test, or two-way ANOVA (for ELISA) *p < 0.05, **p < 0.01; n.a., not applicable; ns, not significant.

To investigate the impact of *Schistosoma* infection on immune responses to COVID-19 mRNA vaccination, C57BL/6J mice were infected with *S. mansoni* (*Sm*) for 8 weeks before the first immunization. *Sm*-infected and non-infected mice were then immunized twice with 1 or 5 µg of mRNA vaccine at 4-week intervals. The final analyses were performed 1 week after the boost immunization. *Sm*-infected and non-infected mice without vaccination served as controls (**Figure 1A**).

Comparable numbers of *Schistosoma* eggs in the livers of *Sm*-infected mice confirmed equivalent infection status across treatment groups (**Figure 1B**). SARS-CoV-2 spike-specific IgG was undetectable in unvaccinated *Sm*-infected and non-infected mice. In contrast, both 1- and 5-µg mRNA vaccinations induced similarly high spike-specific IgG titers in *Sm*-infected and non-infected mice (**Figure 1C**). Notably, vaccinated non-infected mice predominantly produced IgG_2c_, indicating a Th1-skewed response, while vaccinated *Sm*-infected mice exhibited a predominance of IgG_1_ production, consistent with a Th2-biased immune profile in the *Sm*-infected animals (**Figure 1D**).

Beyond antibody responses, mRNA vaccination induced robust spike-specific CD4 and CD8 T-cell responses, as assessed by intracellular cytokine staining (ICS) of splenocytes (**Figures 1E, F**, gating strategies shown in **Supplementary Figure 1A**). In *Sm*-infected mice, mRNA vaccination elicited dose-dependent SARS-CoV-2 spike-specific CD4 T-cell responses (IFNγ^+^ and multifunctional) comparable to or even stronger than those detected in non-infected mice (**Figure 1E**; **Supplementary Figure 1B**). In contrast, spike-specific CD8 T-cell responses were significantly reduced in *Sm*-infected mice compared to non-infected mice (**Figure 1F**, upper panel). *Sm* infection also markedly impaired the polyfunctionality of vaccine-induced CD8 T cells, as evidenced by decreased frequencies of spike-specific IFNγ^+^TNFα^+^ and IFNγ^+^TNFα^+^IL-2^+^ CD8 T-cell subsets (**Figure 1F**, lower panel; **Supplementary Figure 1C**). This was accompanied by an increased proportion of spike-specific PD-1^+^ Lag3^+^ CD8 T cells (**Supplementary Figure 1D**).

Consistent with these findings, splenocytes from *Sm*-infected mice tended to secrete lower amounts of Th1 cytokines (IFNγ, TNFα, and IL-2) following spike protein stimulation (**Figure 1G**), but higher amounts of Th2 cytokines, like spike-specific IL-4, IL-5, and SEA-specific IL-5 and IL-10 (**Figure 1G**; **Supplementary Figure 1E**), although most differences did not reach statistical significance. Both 1- and 5-µg mRNA vaccinations elicited robust SARS-CoV-2 virus neutralizing activity in *Sm*-infected mice, comparable to that observed in non-infected mice (**Figure 1H**). The enhanced Th2 cytokine production likely reflects the persistent Th2-biased immune milieu established during *Sm* infection, allowing for an unaltered humoral response but causing a reduced functionality of SARS-CoV-2 spike-specific CD8 T cells.

### 3.2 Prolonged *Schistosoma* infection does not further impair COVID-19 mRNA vaccine-induced immunity

**Figure 2:**
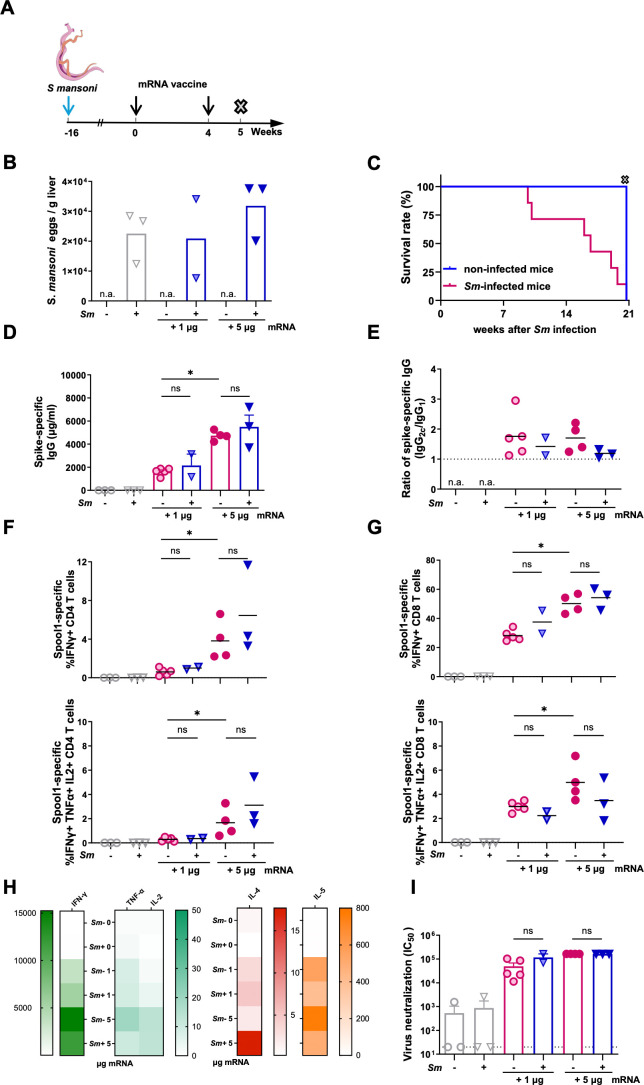
Immunogenicity of mRNA vaccine in mice with 16-week *Schistosoma* infection. C57BL/6J mice were infected subcutaneously with 80 *S. mansoni* cercariae 16 weeks prior to the first immunization. Six mice per group were initially infected; however, by week 16, only two to three *Sm*-infected mice survived. At weeks 0 and 4, surviving *Sm*-infected (*n* ≥ 2) and non-infected mice (*n* ≥ 4) were immunized intramuscularly with 1 or 5 µg of mRNA-based Comirnaty vaccine. *Sm*-infected and non-infected mice (*n* = 3) that did not receive vaccination served as unvaccinated controls. One week after boost immunization (week 5), mice were sacrificed to evaluate vaccine-induced immune responses. **(A)** Schematic depiction of *Schistosoma* infection and vaccination regimen. C57BL/6J mice were infected subcutaneously with 80 *S. mansoni* cercariae 16 weeks prior to the first immunization. Six mice per group were initially infected; however, by week 16, only two to three *Sm*-infected mice survived. At weeks 0 and 4, surviving *Sm*-infected mice (*n* ≥ 2) and non-infected mice (*n* ≥ 4) were immunized intramuscularly with 1 or 5 µg of mRNA-based Comirnaty vaccine. *Sm*-infected and non-infected mice (*n* = 3) that did not receive vaccination served as unvaccinated controls. One week after boost immunization (week 5), mice were sacrificed to evaluate vaccine-induced immune responses. **(B)** The *S. mansoni* egg counts per gram of liver of *Sm*-infected mice. **(C)** The survival rate of *Sm*-infected and non-infected mice during the entire experiment. At week 21 post *Sm* infection, all the leftover mice were sacrificed for final analyses. **(D)** Amounts and **(E)** IgG_2c_/IgG_1_ ratio of SARS-CoV-2 spike-specific IgG antibodies in the sera from vaccinated mice at week 5. **(F, G)** Percentages of spike-specific IFNγ^+^ and IFNγ^+^TNFα^+^IL-2^+^ CD4 **(F)** and CD8 **(G)** T-cell responses determined by ICS of murine splenocytes following stimulation with a spike-specific peptide pool at week 5. **(H)** Cytokines secreted into the supernatants of spike protein stimulated splenocytes within 48 h. Data are presented as fold change compared to non-infected group. **(I)** SARS-CoV-2 virus infection neutralization capacity of the murine sera from the vaccinated mice at week 5. Data are presented as mean ± SEM. Statistical analyses utilized the Mann–Whitney or Kruskal–Wallis test, *p < 0.05; n.a., not applicable; ns, not significant.

Prolonged *Schistosoma* infection can lead to an increased egg burden (28), potentially affecting vaccination responses even further. Thus, instead of 8 weeks, C57BL/6J mice were infected with *Sm* for 16 weeks before the first immunization (**Figure 2A**). Indeed, in these mice, egg burden was markedly increased (**Figure 2B**), while mouse survival was reduced due to progressive hepatic damage and portal-venous inflammation, typically becoming evident by 10–12 weeks post-infection (29) (**Figure 2C**).

Nevertheless, SARS-CoV-2 mRNA vaccinations elicited dose-dependent spike-specific antibody responses in the *Sm*-infected mice, comparable to those in non-infected mice (**Figure 2D**). The IgG subclass distribution showed a similar trend to that observed in **Figure 1**, with non-infected mice exhibiting higher IgG_2c_ responses, while *Sm*-infected mice displayed a more mixed IgG_2c_ and IgG_1_ profile (**Figure 2E**). This confirmed that even prolonged *Sm* infection did not impair humoral vaccine immunogenicity.

Spike-specific CD4 T-cell responses were dose-dependent but comparable in *Sm*-infected and non-infected mice (**Figure 2F**; **Supplementary Figure 2A**). Spike-specific IFNγ^+^ CD8 T-cell responses were also comparable between *Sm*-infected and non-infected mice (**Figure 2G**, upper panel). Multifunctional CD8 T cells (IFNγ^+^TNFα^+^ and IFNγ^+^TNFα^+^IL-2^+^) exhibited only a non-significant trend toward lower frequencies in *Sm*-infected mice, likely due to the limited number of *Sm*-infected mice surviving until the final analysis (**Figure 2G**, lower panel, and **Supplementary Figure 2B**).

Consistent with the T-cell findings by ICS, splenocytes from *Sm*-infected mice secreted lower levels of Th1 cytokines (IFNγ, TNFα, and IL-2) following spike protein stimulation (**Figure 2H**). *Sm*-infected mice also showed a trend toward lower levels of spike-specific Th2 cytokines (IL-4 and IL-5) compared to non-infected mice (**Figure 2H**), and lower SEA-specific Th2 cytokines (IL-5 and IL-10) compared to the 8-week *Sm* infection group (**Supplementary Figures 1E**, **2C**), possibly reflecting overall immunosuppression during the chronic infection phase of *Schistosoma* (28), though these differences were not statistically significant. Finally, despite the higher parasite burden and prolonged infection, both 1- and 5-µg COVID-19 mRNA vaccinations elicited robust SARS-CoV-2 neutralizing activity in *Sm*-infected mice, comparable to that in non-infected mice (**Figure 2I**).

Together, prolonged *Sm* infection increased parasite burden and markedly reduced survival but did not further compromise the humoral or neutralizing antibody response to COVID-19 mRNA vaccination.

### 3.3 *Schistosoma* infection has a minor effect on immune responses to COVID-19 protein vaccination

Apart from mRNA vaccines, recombinant protein-based vaccines represent another central platform developed in response to the COVID-19 pandemic. Given that mRNA and protein vaccines elicit immune responses through distinct mechanisms (30, 31), we next examined how *Sm* infection influences immune responses to a protein-based COVID-19 vaccine. Because prolonged (16-week) *Sm* infection did not further impair vaccine-induced immunity but resulted in a high mortality rate, subsequent experiments were performed by immunization after an 8-week infection.

**Figure 3:**
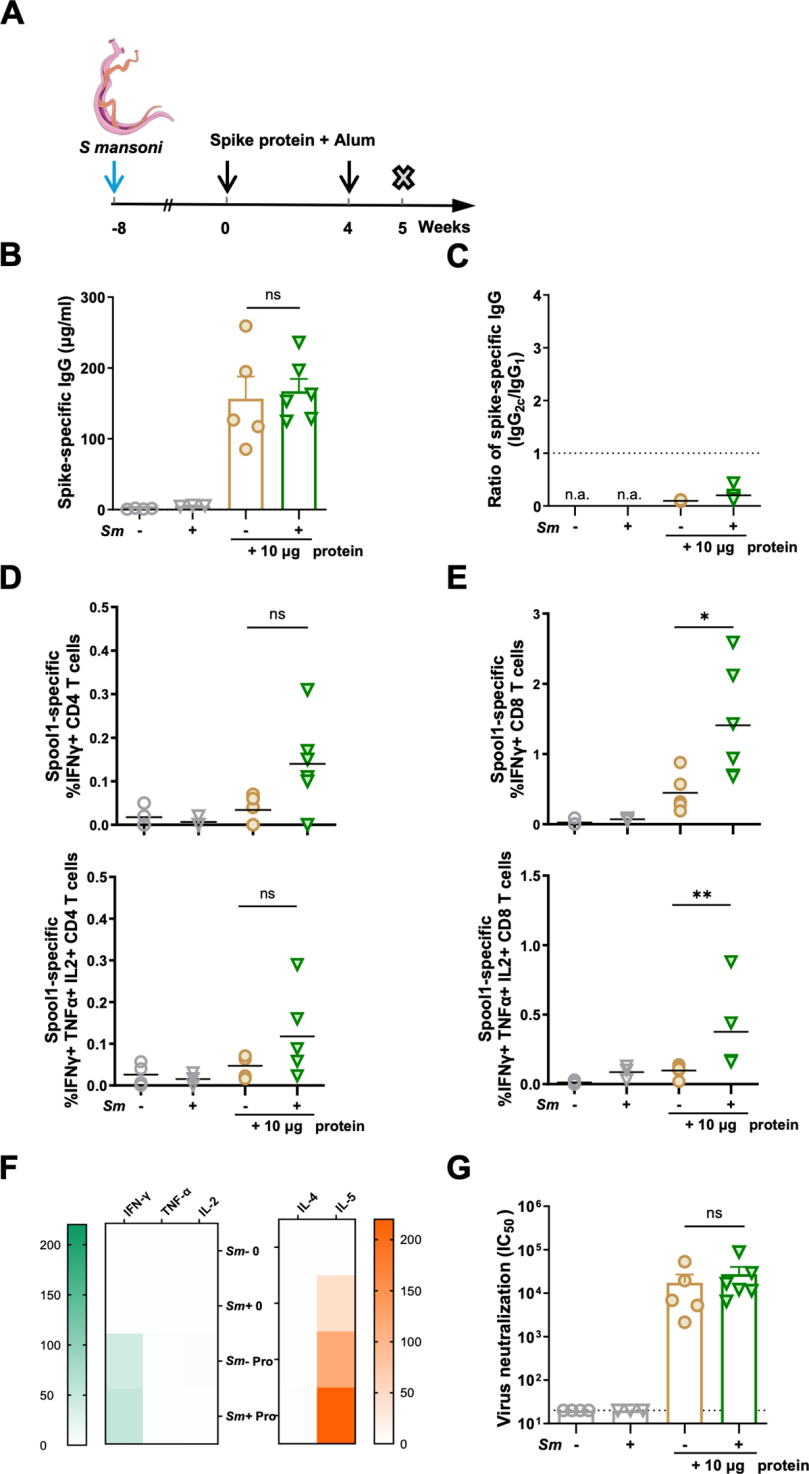
Immunogenicity of spike protein in mice with 8-week *Schistosoma* infection. C57BL/6 mice were infected with 80 *S. mansoni* cercariae subcutaneously 8 weeks before the first immunization. At weeks 0 and 4, *Sm*-infected and non-infected mice (*n* ≥ 5) were immunized intramuscularly with 10 µg of alum-adjuvanted spike protein vaccine. *Sm*-infected and non-infected mice (*n* = 3) without vaccination served as controls. One week after boost immunization (week 5), mice were sacrificed to evaluate vaccine-induced immune responses. **(A)** Schematic depiction of *Schistosoma* infection and vaccination regimen. C57BL/6 mice were infected with 80 *S. mansoni* (Sm) cercariae subcutaneously 8 weeks before the first immunization. At weeks 0 and 4, *Sm*-infected and non-infected mice (*n* ≥ 3) were immunized intramuscularly with 10 µg of spike protein + Alum. *Sm*-infected and non-infected mice (*n* = 5) without vaccination served as controls. One week after boost immunization (week 5), mice were sacrificed to evaluate vaccine-induced immune responses. **(B)** Amounts and **(C)** IgG_2c_/IgG_1_ ratio of SARS-CoV-2 spike-specific IgG antibodies in the sera from vaccinated mice at week 5. **(D, E)** Percentages of spike-specific IFNγ^+^ and IFNγ^+^TNFα^+^IL-2^+^ CD4 **(D)** and CD8 **(E)** T-cell responses determined by ICS of murine splenocytes following stimulation with a spike-specific peptide pool at week 5. **(F)** Cytokines secreted into the supernatants of spike protein stimulated splenocytes within 48 h. Data are presented as fold change compared to the non-infected group. **(G)** SARS-CoV-2 virus infection neutralization capacity of the murine sera from the vaccinated mice. Data are presented as mean ± SEM. Statistical analyses utilized the Mann–Whitney or Kruskal–Wallis test, *p < 0.05; n.a., not applicable; ns, not significant.

*Sm*-infected and non-infected mice were immunized twice at a 4-week interval with a trimeric SARS-CoV-2 spike protein vaccine formulated with alum as adjuvant (**Figure 3A**). *Sm*-infected and non-infected mice without vaccination served as controls. Trimeric spike vaccination induced comparable antibody titers in *Sm*-infected and non-infected mice (**Figure 3B**). The IgG subclass analysis revealed IgG_1_ as the predominant subclass in both groups, likely reflecting the property of the alum adjuvant to skew immune responses toward a Th2-profile (**Figure 3C**). These findings indicate that *Schistosoma* infection had minimal effects on the magnitude or subtype of IgG antibody responses elicited by an alum-adjuvanted protein vaccination.

As expected, alum-adjuvanted protein vaccination elicited spike-specific CD4 T cells, albeit at a significantly lower level than the mRNA vaccine, with *Sm* mice exhibiting a slightly stronger response than non-infected mice (**Figure 3D**; **Supplementary Figure 3A**). Surprisingly, detectable spike-specific CD8 T-cell frequencies and functional properties were increased in *Sm*-infected mice, including spike-specific IFNγ^+^ and multifunctional IFNγ^+^TNFα^+^ and IFNγ^+^TNFα^+^IL-2^+^ CD8 T cells (**Figure 3E**; **Supplementary Figure 3B**). Cytokine profiling of splenocytes following spike protein stimulation mirrored the T-cell findings, as *Sm*-infected mice secreted higher levels of both Th1- and Th2-associated cytokines in response to spike and SEA stimulation compared with non-infected mice (**Figure 3F**; **Supplementary Figure 3C**), though these differences were not statistically significant.

Finally, both *Sm*-infected and non-infected mice developed comparable SARS-CoV-2 neutralizing activity following protein vaccination (**Figure 3G**), indicating that *Schistosoma* infection had no impact on vaccine-induced CD4 T-cell and antibody responses that were able to neutralize SARS-CoV-2 and even promoted spike-specific CD8 T-cell responses.

### 3.4 COVID-19 mRNA vaccination elicits stronger immune responses than protein vaccination in *Schistosoma*-infected mice

**Figure 4:**
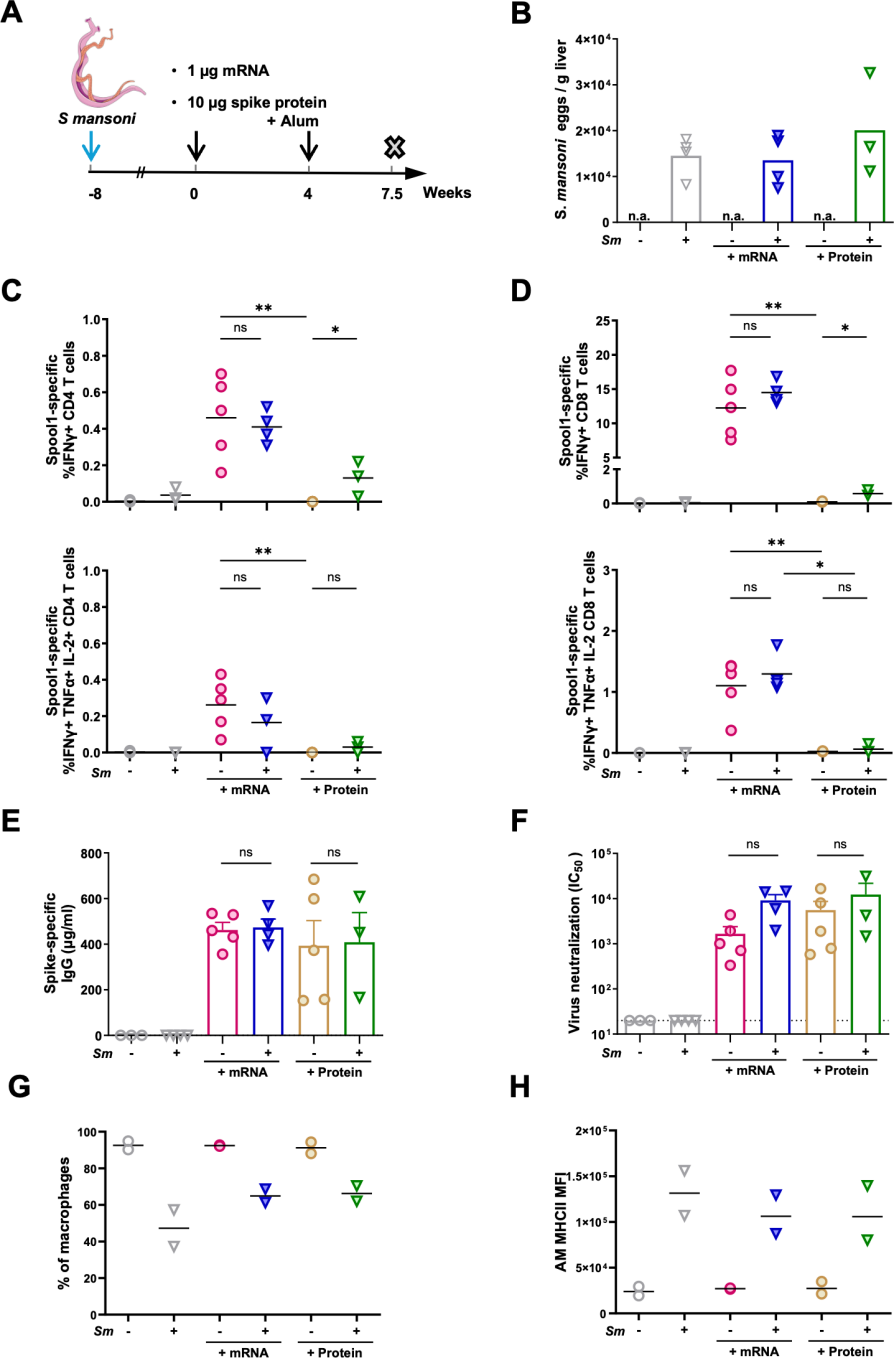
Immunogenicity comparison of mRNA and spike protein vaccines in mice with *Schistosoma* infection. **(A)** Schematic depiction of *Schistosoma* infection and vaccination regimen. C57BL/6 mice were infected with 80 *Sm* Cercariae subcutaneously 8 weeks before the first immunization. At weeks 0 and 4, *Sm*-infected and non-infected mice (*n* ≥ 3) were immunized intramuscularly with 1 µg of mRNA-based Comirnaty or 10 µg of alum-adjuvanted spike protein vaccine. *Sm*-infected and non-infected mice (*n* = 3) without vaccination served as controls. After 3.5 weeks of boost immunization (week 7.5), mice were sacrificed to evaluate vaccine-induced immune responses. **(B)** The *S. mansoni* egg counts per gram liver of *Sm*-infected mice. **(C, D)** Percentages of spike-specific IFNγ^+^ and IFNγ^+^TNFα^+^IL-2^+^ CD4 **(C)** and CD8 **(D)** T-cell responses determined by ICS of murine splenocytes following stimulation with a spike-specific peptide pool at week 7.5. **(E)** Amounts of SARS-CoV-2 spike-specific IgG antibodies in the sera from vaccinated mice. **(F)** SARS-CoV-2 virus infection neutralization capacity of the murine sera from the vaccinated mice. **(G)** Percentages of CD64^+^ F4/80^+^ CD11c^+^ lung macrophages within the total macrophage compartment (CD64^+^ F4/80^+^). **(H)** Mean fluorescence intensity (MFI) of MHC-II expression by CD64^+^ F4/80^+^ CD11c^+^ lung macrophages. G and H analyzed by flow cytometry from pooled samples in two independent experiments. Statistical analyses utilized the Mann–Whitney or Kruskal–Wallis test, *p < 0.05, **p < 0.01; n.a., not applicable; ns, not significant.

Next, we compared the immunogenicity of mRNA and protein vaccination. To facilitate the estimation of protective immune responses in the next step, we compared antibody and T-cell responses 3.5 weeks after the last vaccination (**Figure 4A**). Non-infected and *Sm*-infected mice with comparable infection levels (**Figure 4B**) were immunized with either 1 µg of mRNA or 10 µg of alum-adjuvanted recombinant spike protein vaccine.

mRNA vaccination induced significantly stronger spike-specific CD4 and CD8 T-cell responses than protein vaccination in both non-infected and *Sm*-infected mice. Spike-specific IFNγ^+^ and IFNγ^+^TNFα^+^IL-2^+^ CD4 T-cell responses were readily detectable in the mRNA-vaccinated mice, whereas much lower responses were observed following protein vaccination (**Figure 4C**). Interestingly, *Sm* infection enhanced IFNγ^+^ CD4 T-cell responses after protein vaccination, reaching levels similar to those observed at the earlier 1-week time point (**Figure 3C**). mRNA vaccination also generated robust IFNγ^+^ and multifunctional IFNγ^+^TNFα^+^IL-2^+^ CD8 T-cell responses in both groups of mice (**Figure 4D**). Unlike at earlier time points, suppression previously associated with *Sm* infection was no longer evident 3.5 weeks after vaccination, and CD8 T-cell functionality had become comparable between *Sm*-infected and non-infected mice (**Figure 4D**). In contrast, protein vaccination only induced weak CD8 T-cell responses, detectable exclusively in *Sm*-infected mice. As expected, T-cell responses from both vaccine groups 3.5 weeks after boost vaccination were weaker than those observed 1 week after immunization (**Figures 1**-**3**), indicating a contraction of vaccine-induced cellular immunity over time.

Spike-specific antibody titers and SARS-CoV-2 neutralizing activity were also comparable between mRNA- and protein-vaccinated groups at this later time point, indicating that both vaccine platforms maintained similar long-term humoral immunity (**Figures 4E, F**).

To assess how *Sm* infection alters antigen presentation in the lung in our model, we profiled CD64^+^ F4/80^+^ CD11c^+^ lung macrophages, which, despite forming a lower percentage of the CD64^+^ F4/80^+^ macrophage compartment (**Figure 4G**), consistently displayed increased MHC-II and CD86 expression in the *Sm*-infected mice (**Figure 4H**; **Supplementary Figure 4A**, **5**). Analysis of dendritic cell subsets revealed a reduction in the abundance of classical type 1 dendritic cells (cDC1) and a shift toward cDC2 dominance in infected mice, accompanied by increased expression of CD86 on cDC2 (**Supplementary Figures 4B–F**). These effects were already evident at 7 weeks post-infection, i.e., prior to the initiation of immunization (**Supplementary Figures 4G, H**).

Together, these findings show that the COVID-19 mRNA vaccine induces stronger and more multifunctional T-cell responses than the protein vaccine in both non-infected and *Sm*-infected mice. Nonetheless, in the longer-term setting, both types of vaccines maintained comparable spike-specific antibody and virus-neutralizing activities, indicating that durable humoral immunity is largely independent of helminth infection or the type of vaccine used.

### 3.5 COVID-19 mRNA and spike protein vaccines confer comparable protection against SARS-CoV-2 challenge in *Schistosoma*-infected mice

**Figure 5:**
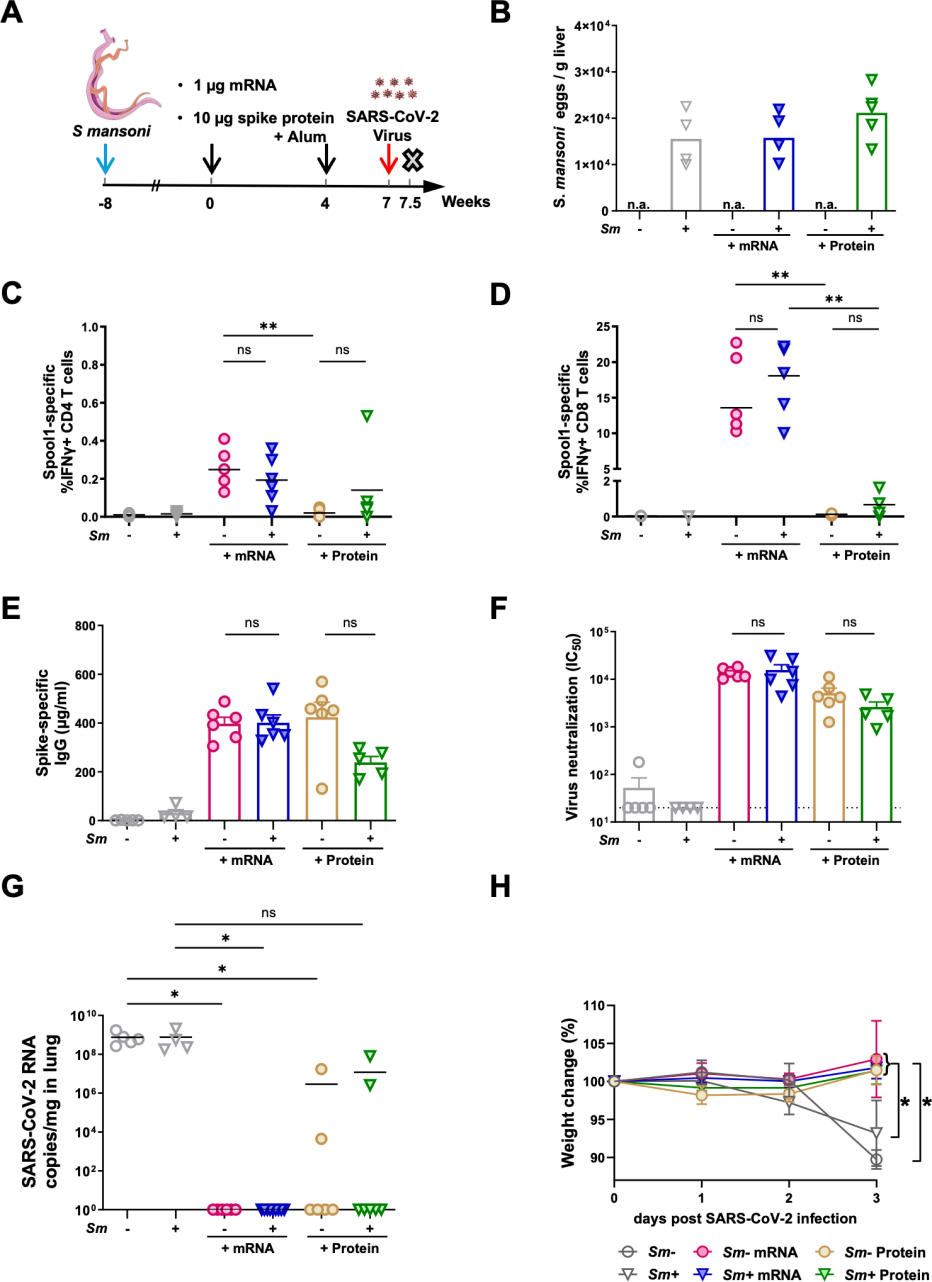
Protective efficacy of mRNA and spike protein vaccines in *Schistosoma*-infected mice after SARS-CoV-2 challenge. **(A)** Schematic depiction of experimental setup. After the establishment of *Schistosoma* infection, at weeks 0 and 4, *Sm*-infected and non-infected mice (*n* ≥ 5) were immunized intramuscularly with 1 µg of mRNA-based Comirnaty or 10 µg of alum-adjuvanted spike protein. *Sm*-infected and non-infected mice (*n* ≥ 4) without vaccination served as controls. Three weeks after boost immunization (week 7), the mice were aerosol-inoculated with 5 × 10^5^ PFU mouse-adapted SARS-CoV-2 MA20 strain in a total volume of 6 mL. Three days after SARS-CoV-2 virus challenge, mice were sacrificed for the final analyses. **(B)** The *Sm* egg counts per gram liver of *Sm*-infected mice. **(C, D)** Percentages of spike-specific IFNγ^+^ CD4 **(C)** and CD8 **(D)** T-cell responses determined by ICS of murine splenocytes following stimulation with a spike-specific peptide pool. **(E)** Amounts of SARS-CoV-2 spike-specific IgG antibodies in the sera from vaccinated mice. **(F)** SARS-CoV-2 virus infection neutralization capacity of the murine sera from the vaccinated mice. **(G)** SARS-CoV-2 RNA copies per microgram of lung tissues of infected mice. **(H)** Daily percent weight change of SARS-CoV-2 infected mRNA- and protein-vaccinated mice compared to initial weight. Significance between groups of weight change was assessed at day 3 post viral infection. Statistical analyses utilized the Mann–Whitney or Kruskal–Wallis test **(C–F)**, two-way ANOVA **(G)**, *p < 0.05, **p < 0.01; n.a., not applicable; ns, not significant.

In the next step, we evaluated potential differences in protective efficacy between the mRNA and protein vaccines. Non-infected and *Sm*-infected mice with comparable helminth infection levels were challenged with the mouse-adapted SARS-CoV-2 MA20 strain 3 weeks after the final immunization (**Figures 5A, B**). Immunological and virological analyses were performed 3 days after SARS-CoV-2 infection.

After viral challenge, and consistent with the immune profiles prior to infection described in **Figure 4**, mRNA-vaccinated animals exhibited strong spike-specific CD4 and CD8 T-cell responses, whereas T-cell activation was barely detectable in the protein-vaccinated groups (**Figures 5C, D**). Importantly, no significant differences were detected between non-infected and *Sm*-infected mice within each vaccine group, indicating that *Schistosoma* infection did not impair the recall of vaccine-induced cellular immunity following exposure to SARS-CoV-2.

Consistent with these findings, both vaccine groups mounted robust humoral recall responses after infection. Although spike-specific IgG antibody titers tended to be lower in protein-vaccinated, *Sm*-infected mice, neutralizing antibody activity remained high (**Figures 5E, F**) and was comparable between non-infected and *Sm*-infected mice within each vaccine group, confirming that *Schistosoma* infection did not alter the magnitude or quality of recall humoral immunity.

Quantification of viral RNA in lung tissues by qPCR showed complete viral clearance in all mRNA-vaccinated mice irrespective of *Sm* infection status, whereas high SARS-CoV-2 levels were detected in unvaccinated controls (**Figure 5G**). In contrast, SARS-CoV-2 RNA was detectable in two mice from each protein-vaccinated group, corresponding to protection in five of seven animals (**Figure 5G**). These results are consistent with the stronger T-cell responses and more uniform neutralizing antibody titers elicited by the mRNA vaccine. Moreover, vaccinated animals maintained stable body weight, whereas non-vaccinated controls exhibited a marked (5%–10%) weight loss already shortly after SARS-CoV-2 infection (**Figure 5H**).

**Figure 6:**
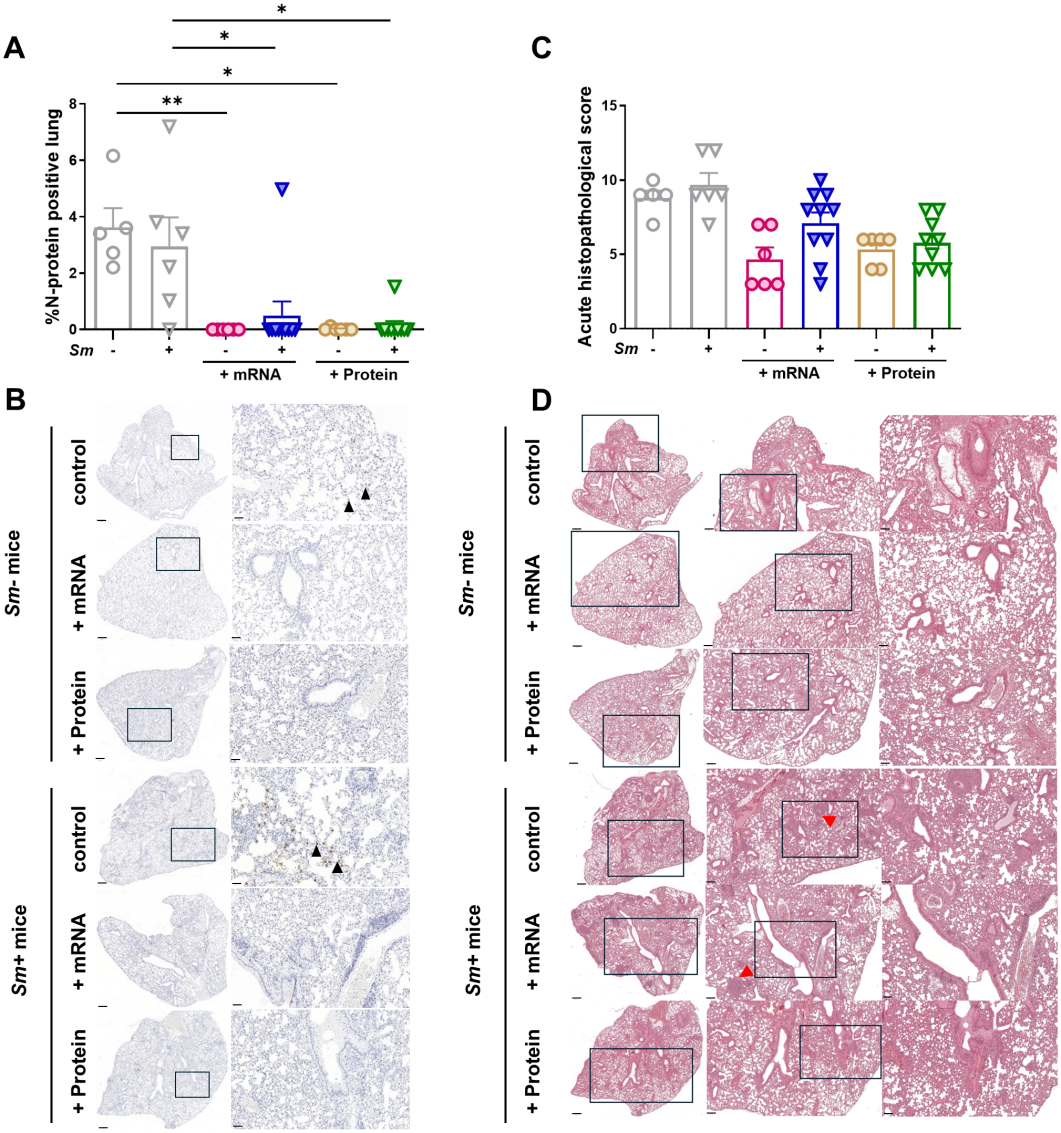
Lung histopathology in *Schistosoma*-infected mice following SARS-CoV-2 challenge after mRNA or protein vaccination. **(A)** Percentages of SARS-CoV-2–positive cells by immunohistology staining of lung tissues. Data are presented as mean ± SEM. **(B)** Immunohistological staining (IHC) of lung tissue for SARS-CoV-2 nucleocapsid from non-infected and *Sm* mice following SARS-CoV-2 infection after mRNA or protein vaccination. Patchy antigen staining in non-vaccinated, non-infected, or *Sm*-infected mice in damaged lung parenchyma (black arrowheads); vaccinated groups show no positive antigen staining. Scale bars: 400/500 µm (low), 200 µm (middle), and 50 µm (high). **(C)** Histopathological scoring of lung tissue. Composite acute viral histopathology score based on bronchiolitis, interstitial pneumonia, and alveolar damage, each scored 0–4 based on the severity of the injury. **(D)** Hematoxylin and eosin (H&E) staining of lung tissue from *Sm* mice following SARS-CoV-2 infection after mRNA or protein vaccination. Helminth-related eggs and granulomas may be present in all groups (red arrowheads). Scale bars: 400/500 µm (low), 200 µm (middle), 50 µm (high). Statistical analyses utilized the Kruskal–Wallis test, *p < 0.05, **p < 0.01.

To assess whether these findings correlated with lung tissue damage, we evaluated the immunohistochemistry and histopathology of lung tissue sections from different treatment groups. Here, correlating to the viral load determined by qPCR (**Figure 5G**), lungs from vaccinated mice stained negative for viral antigen, whereas unvaccinated mice showed patchy SARS-CoV-2 N-antigen expression in damaged parenchymal and bronchiolar epithelium (**Figures 6A, B**). Quantification using a composite acute viral histopathology score (bronchiolitis/bronchitis, interstitial pneumonia, and alveolar damage/exudate) revealed acute viral injury in non-vaccinated mice (**Figures 6C, D**), whereas vaccinated mice exhibited low parenchymal viral injury.

These data demonstrate that both COVID-19 mRNA and protein vaccines provided effective protection against SARS-CoV-2 infection in *Sm*-infected mice. Despite remarkable differences in the magnitude of CD4 and CD8 T-cell responses, the two vaccine platforms achieved comparable control of viral replication and protected from lung pathology, although the protein vaccine group exhibited greater inter-individual variation. Notably, *Sm* infection did not diminish vaccine-induced viral protection.

## 4 Discussion

In this study, we investigated whether chronic helminth infection affects the effectiveness of COVID-19 vaccination in mice. COVID-19 mRNA and alum-adjuvanted trimeric spike protein vaccines elicited comparable antibody responses in *Sm*-infected and non-infected mice. *Sm* infection had little impact on T-cell responses to protein vaccination, which were overall weaker than those elicited by the mRNA vaccine. In contrast, mRNA vaccination during the Th2 infection phase resulted in significantly reduced numbers and function of vaccine-specific CD8 T cells. Neutralizing antibody titers were comparable after mRNA and protein vaccination against SARS-CoV-2 in *Sm*-infected and non-infected mice. However, mRNA vaccination enabled a stronger recall response after virus challenge, resulting in protective efficacy against SARS-CoV-2 infection, irrespective of the underlying *Sm* infection.

Interestingly, *Sm* infection did not alter the course of SARS-CoV-2 coinfection, which contrasts with our recent findings with experimental hepatitis B virus infection, where *Schistosoma*-induced IFNγ led to strong suppression of viral replication in the liver (21). This highlights the importance of compartmentalization when studying the effects of co-infection in the context of vaccine efficacy.

A key observation was that prolonged *Sm* infection did not further suppress CD8 T-cell responses to mRNA vaccination. Consistent with this, IL-10 release was also higher in mice vaccinated at 8 weeks post-infection compared to 16 weeks. This aligns with mechanistic work demonstrating that helminth-mediated IL-10 signaling can blunt vaccine-induced T-cell immunity and that IL-10 blockade rescued CD8 T-cell responses (17). SEA-specific IL-10 during *Sm* infection has been shown to rise shortly after the onset of egg deposition, peak between 5 and 10 weeks, and remain elevated throughout chronic infection up to at least 29 weeks (32, 33). These data support a model in which the IL-10-dominated regulatory environment capable of suppressing antiviral T-cell responses is already established by week 8 and does not intensify with infection duration. Consequently, the suppressive impact on vaccine-induced immunity appears to be a threshold effect established early in the chronic phase, rather than a cumulative process that intensifies with prolonged parasite exposure.

Vaccine platform-specific differences under *Schistosoma* infection were also evident. SARS-CoV-2 mRNA vaccines are strongly dependent on Th1 responses and the cytotoxic CD8 T-cell priming pathway (34), which are attenuated during SARS-CoV-2 infection. In contrast, alum preferentially drives Th2 and antibody-biased responses (35), which are largely preserved in a helminth-induced Th2 milieu. This divergence was also evident in the populations of antigen-presenting cells. In *Sm*-infected mice, we observed a shift from cDC1 to cDC2 populations. CD8 T-cell priming after mRNA vaccination normally depends on cDC1-mediated cross-presentation (36); however, in the lung, cDC2 has been shown to be sufficient for CD8 T-cell priming via cross-presentation (37). Modified antigen presentation is also evident in *Schistosoma-*infected humans, with a shift toward cDC2 populations in the lung evident from 2 weeks post-infection (38).

Although *Schistosoma-*infected mice exhibited reduced frequencies and function of CD8 T cells, recall expansion after viral challenge remained intact. Even modest priming can yield strong recall responses during infection (39–44), and human studies similarly show enhanced recall in infected-then-vaccinated individuals compared to those only vaccinated (45), with recall also observed despite CD8 T-cell exhaustion markers upregulated (46, 47).

Despite these immunological alterations, both vaccines conferred robust protection against SARS-CoV-2 challenge, with consistently better protection following mRNA vaccination. Neutralizing antibodies and CD4 T cells are maintained at high levels, and these processes are not impeded by the type 2 response to *Schistosoma* infection. In contrast, other effects of helminth infection, such as increased activation of lung macrophages, can also contribute to protection (48). This supports previous studies in humans and rhesus macaques, which have demonstrated that even low titers of neutralizing antibodies are sufficient for protection against SARS-CoV-2 (49, 50). However, this contrasts with other studies using murine-specific helminth species, which have reported reduced immunogenicity and protection against influenza (18) and SARS-CoV-2 (17) vaccines. A key distinction is that *S. mansoni* undergoes a lung passage and egg deposition that induces focal inflammation and antigen-presenting cell activation (38, 51), which could preserve components of antiviral immunity. *H. polygyrus* is a potent immunomodulator that directly drives regulatory T-cell conversion using a secreted TGF-β mimic (52) and modulates dendritic cell function, including reduced CD86 and MHC-II expression (53). This reduced dendritic cell activation and antigen presentation may impair effective T-cell priming following vaccination, contributing to the more pronounced suppression of cellular immune responses observed in nematode infection models. In contrast, *S. mansoni* infection generates a mixed Th2 and regulatory environment but also sustained granulomatous inflammation, eosinophilia, and activation of tissue macrophages and dendritic cells (54–56). These differences highlight that helminth species exert distinct immunomodulatory effects, with intestinal nematodes often inducing stronger systemic regulatory responses, whereas *S. mansoni* infection is characterized by a mixed Th2 and inflammatory environment, particularly due to egg deposition in tissues. This may explain why vaccine-induced protection is preserved in our model despite modulation of cellular immunity.

Evidence from human studies supports this pattern of helminth-modulated but not abolished vaccine immunity. A recent systematic review and meta-analysis reported that helminth infection consistently reduces vaccine-induced cellular responses, while antibody titers were often preserved (12). In an Ugandan cohort, individuals with *S. mansoni* achieved protective antibody levels following tetanus and hepatitis B vaccination, although titers declined more rapidly, and cellular responses were attenuated (57). For SARS-CoV-2, moderate-to-heavy *Sm* infection was associated with reduced anti-spike IgG following mRNA vaccination (58). In contrast, lymphatic filariasis did not impair anti-spike or neutralizing responses after viral vector vaccination (59), highlighting species- and platform-specific effects. Together, these findings suggest that helminths can modulate vaccine immunogenicity without necessarily compromising protective efficacy, consistent with our findings.

However, the immune modulation elicited by schistosomiasis is heterogeneous across tissues and infection intensities and may differ in humans with comorbidities or repeated praziquantel exposure. In addition, the mouse-adapted SARS-CoV-2 model used here does not fully recapitulate human infection, and differences in viral entry, tissue tropism, and disease progression compared to transgenic models such as K18-hACE2 mice should be considered when interpreting these findings. Future work should address whether deworming or other vaccination strategies can enhance cellular responses without compromising protection in helminth-endemic populations. Despite these limitations, our findings suggest that SARS-CoV-2 vaccines are likely to remain effective in helminth-endemic regions, while highlighting the importance of including such populations in vaccine immunogenicity studies.

## Data Availability

The raw data supporting the conclusions of this article will be made available by the authors, without undue reservation.
