# Supplementary material for: Helminth infection modulates the immunogenicity of COVID-19 vaccines in mice without compromising protective efficacy
Source: Front Immunol. 2026 May 28;17:1827532. doi: 10.3389/fimmu.2026.1827532 (PMC13252778; doi:10.3389/fimmu.2026.1827532)
Supplement: Supplementary file 1 [file DataSheet1.docx]

***Supplementary Material***

1. **Supplementary Methods**

**1.1 Production and staining of peptide–MHC (pMHC) multimers.**

pMHC monomers were generated as previously described using the FLEXamer system for subsequent biotinylation (1-3). Briefly, recombinantly expressed murine MHC class I heavy chain and murine β2-microglobulin were refolded in the presence of the spike S538 peptide (CVNFNFNGL; Peptides & Elephants, Germany). Properly folded pMHC complexes were purified by size-exclusion chromatography. Site-specific biotinylation was achieved by tubulin tyrosine ligase (TTL)–mediated ligation of 3-azido-L-tyrosine to the tub-tag at the C-terminus of the pMHC molecule, followed by click-chemistry–mediated conjugation to DBCO-PEG4-Biotin (Jena Bioscience, Germany). Biotinylated pMHC monomers were subsequently multimerized using APC-conjugated streptavidin (Invitrogen, USA) at optimized molar ratios to generate pMHC multimers.

For staining, single-cell splenocyte suspensions were incubated with pMHC multimers on ice in the dark for 30 min. Without intermediate washing, cells were subsequently surface-stained with antibodies against CD4, CD8, PD-1, and LAG-3 on ice in the dark for an additional 20 min. Dead cells were excluded using Fixable Viability Dye eF780 (eBioscience, Germany). After a final wash, data were acquired on a CytoFlex S flow cytometer (Beckman Coulter, USA) and analyzed using FlowJo software v10.8 (Tree Star, USA).

1. **Supplementary Figures**


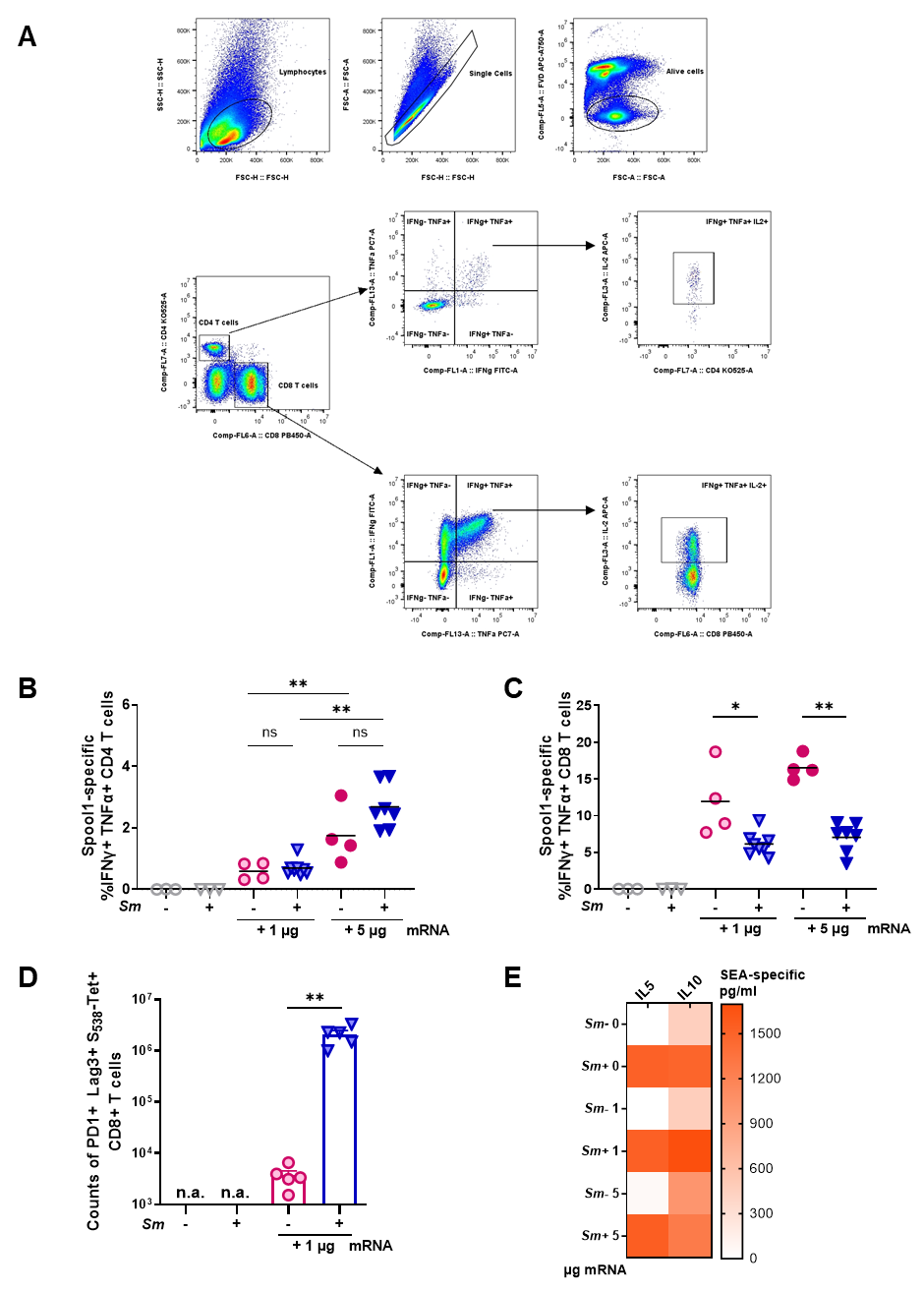


**Supplementary Figure 1 Gating strategy of flow cytometric analysis, spike- and SEA-specific immune responses in mRNA vaccinated mice with 8-week Schistosoma infection.** C57BL/6 mice were infected with 80 *S. mansoni* (*Sm*) Cercariae subcutaneously 8 weeks before 1^st^ immunization. At week 0 and week 4, *Sm*-infected and non-infected mice were immunized intramuscularly with 1 µg or 5 µg mRNA-based Comirnaty vaccine. *Sm*-infected and non-infected mice without vaccination served as controls. 1 week after boost immunization (week 5), mice were sacrificed to evaluate vaccine-induced immune responses. (A) Gating strategy of flow cytometric analysis. The forward- vs sides-scattered-light plot was used to exclude cell debris and gate lymphocyte population (1^st^ diagram). Single cells were selected from lymphocyte population by plotting height vs area of forward-scattered-light (FSC-H vs FSC-A) and gating on the diagonal population (2^nd^ diagram). Alive cells from single cell population were defined by exclusion of the fixable viability dye (FVD)-APC-A750-positive cells (3^rd^ diagram). In the second step, cytokine-positive CD4 and CD8 T-cells. CD4 or CD8 T-cells were gated by plotting CD4-APC against CD8-PB450 within viable lymphocyte population. Right upper panels: IFNγ+ TNFα+ CD4 T-cells were defined by gating IFNγ-FITC against TNFα-PECy7 under CD4 T-cells. Afterwards, IFNγ+TNFα+IL-2+ CD4 were defined by gating IL2-PE against CD4-APC under IFNγ+TNFα+ CD4 T-cells. Right lower panels: IFNγ+TNFα+ CD8 T-cells were defined by gating IFNγ-FITC against TNFα-PECy7 under CD8 T-cells. Afterwards, IFNγ+TNFα+IL-2+ CD8 were defined by gating IL2-PE against CD8-PB450 under IFNγ+TNFα+ CD8 T-cells. The control stimulated with ovalbumin (OVA)-derived peptide was used to set up the gate position. APC, allophycocyanin; PB450, Pacific Blue 450; FITC, fluorescein isothiocyanate; PE, Phycoerythrin; PECy7, PE-Cyanine7. (B, C) Percentages of spike-specific IFNγ+TNFα+ CD4 (B) and CD8 (C) T-cell responses determined by intracellular cytokine staining (ICS) of the splenocytes from 8-week *Sm*-infected mice. (D) Counts of PD1+ Lag3+ S_538_-Tetramer+ CD8 T-cells. (E) The concentration (pg/ml) of IL-5 and IL-10 as measured by ELISA from supernatants of *Sm* egg antigen (SEA)-stimulated splenocytes (48h). Data are presented as mean ± SEM. Statistical analyses utilized the Mann-Whitney or Kruskal-Wallis test, **p*<0.05, ***p*<0.01, ns, not significant.


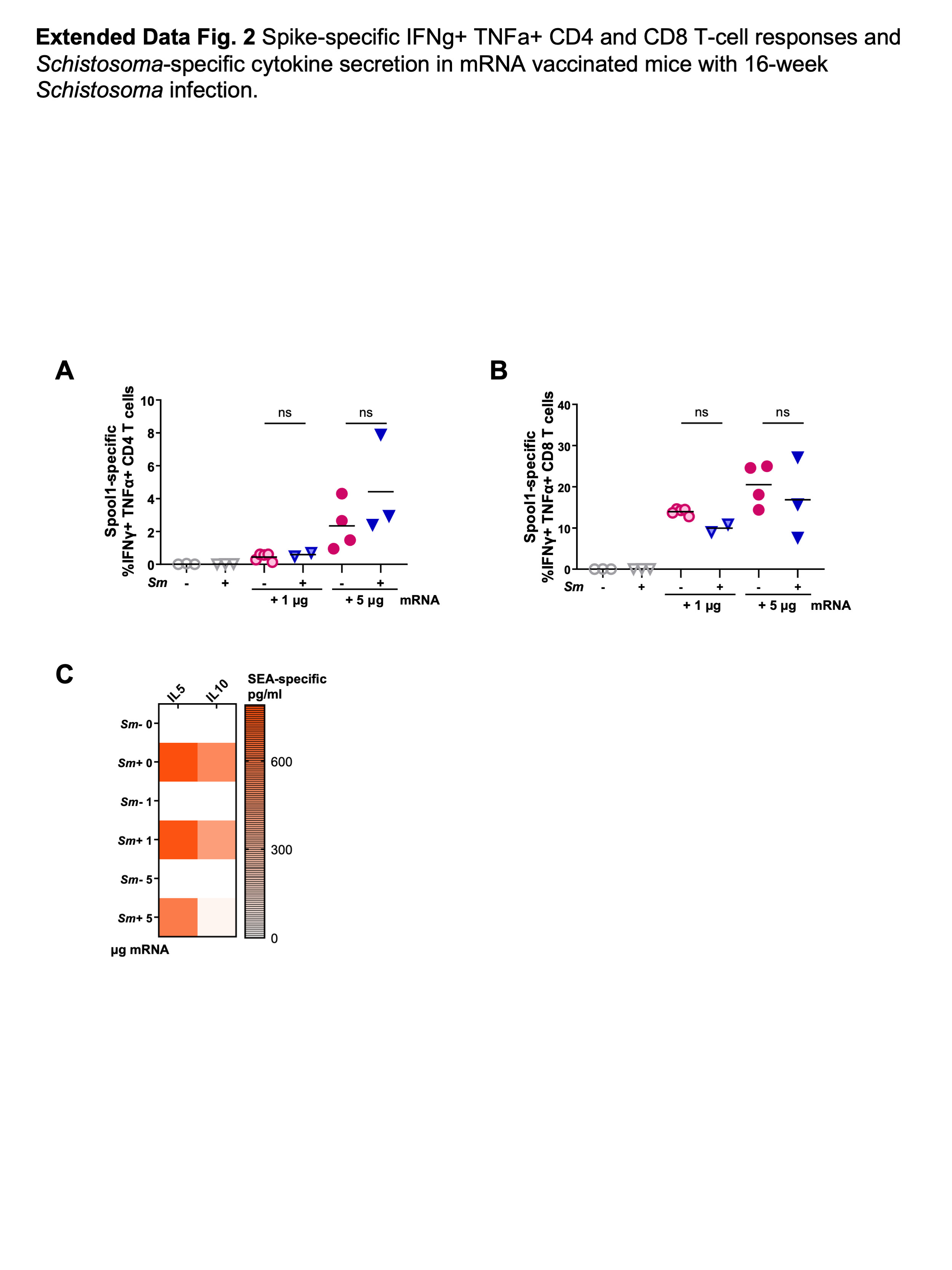


**Supplementary Figure 2 Spike- and *Schistosoma*-specific CD4 and CD8 T-cell responses in mRNA vaccinated mice with 16-week Schistosoma infection.** (A, B) Percentages of spike-specific IFNγ+TNFα+ CD4 (A) and CD8 (B) T-cell responses determined by ICS of the splenocytes from 16-week *Sm*-infected mice. (C) The concentration (pg/ml) of IL-5 and IL-10 as measured by ELISA from supernatants of SEA-stimulated splenocytes (48h). Data are presented as mean ± SEM. Statistical analyses utilized the Mann-Whitney or Kruskal-Wallis test, **p*<0.05, ***p*<0.01, ns, not significant.


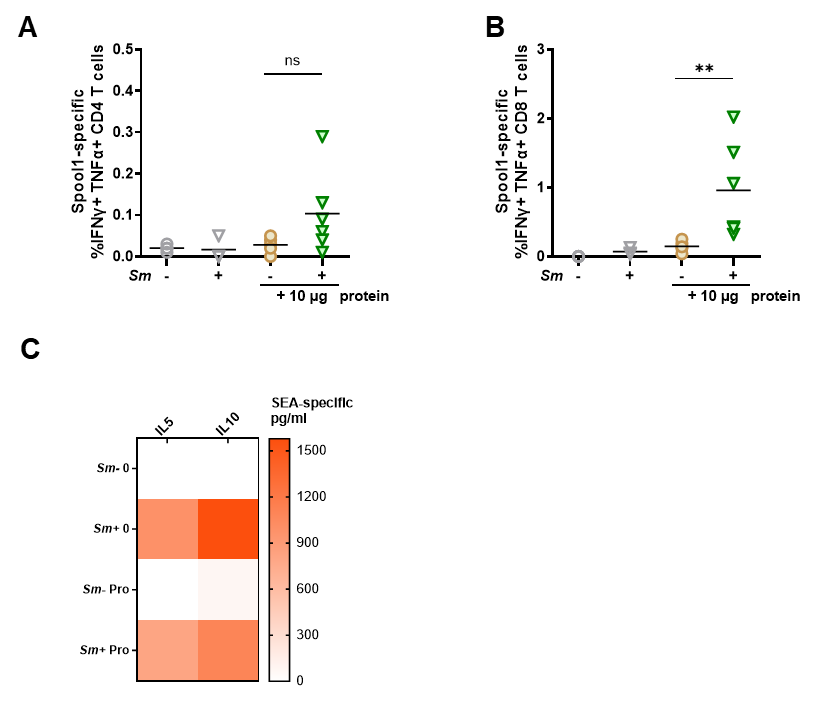


**Supplementary Figure 3 Spike- and *Schistosoma*-specific CD4 and CD8 T-cell responses in protein immunized mice with 8-week *Schistosoma* infection.** C57BL/6 mice were infected with 80 *Sm* Cercariae subcutaneously 8 weeks before 1^st^ immunization. At week 0 and week 4, *Sm*-infected and non-infected mice were immunized intramuscularly with alum adjuvanted 10 µg spike protein vaccine. *Sm*-infected and non-infected mice without vaccination served as controls. 1 week after boost immunization (week 5), mice were sacrificed to evaluate vaccine-induced immune responses. (A, B) Percentages of spike-specific IFNγ+TNFα+ CD4 (A) and CD8 (B) T-cell responses determined by ICS of murine splenocytes following stimulation with a spike-specific peptide pool. (C) The concentration (pg/ml) of IL-5 and IL-10 as measured by ELISA from supernatants of SEA-stimulated splenocytes (48h). Data are presented as mean ± SEM. Statistical analyses utilized the Mann-Whitney or Kruskal-Wallis test, **p*<0.05, ***p*<0.01, n.a., not applicable, ns, not significant.


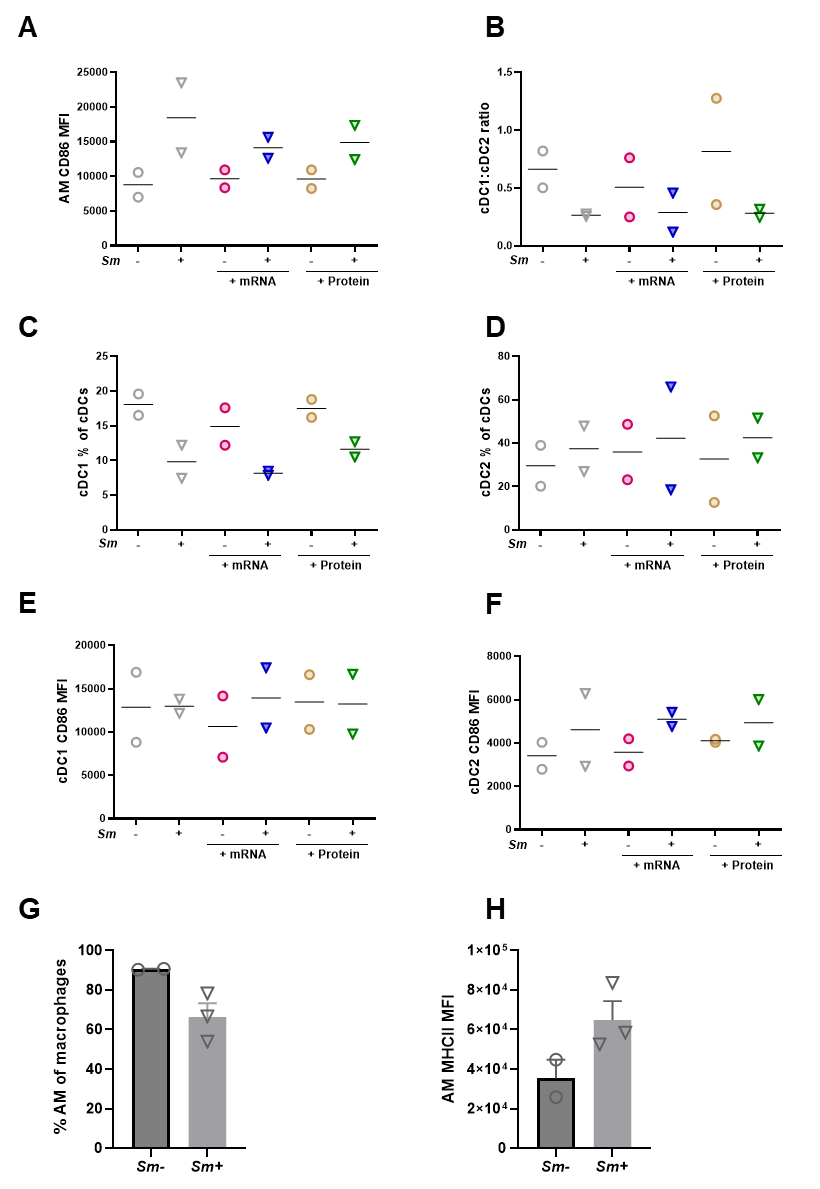


**Supplementary Figure 4 Comparison of antigen presenting cell populations after mRNA and spike protein vaccines in mice with *Schistosoma* infection.** (A) CD86 expression (MFI) by CD11c+ lung macrophages (CD64⁺ F4/80⁺ CD11c⁺). (B) Ratio of lung cDC1:cDC2 cells. (C) Percentage of cDC1 cells in cDC compartment. (D) Percentage of cDC2 cells in cDC compartment. (E) CD86 expression (MFI) by cDC1 cells (F) CD86 expression (MFI) by cDC2 cells. A-F: cells analyzed by flow cytometry from pooled samples in two independent experiments. (G and H) Percentage of CD11c+ lung macrophages from macrophage compartment (G) and MHCII expression (MFI) (H) by the same cells in mice infected for 7 weeks with *S. mansoni*.


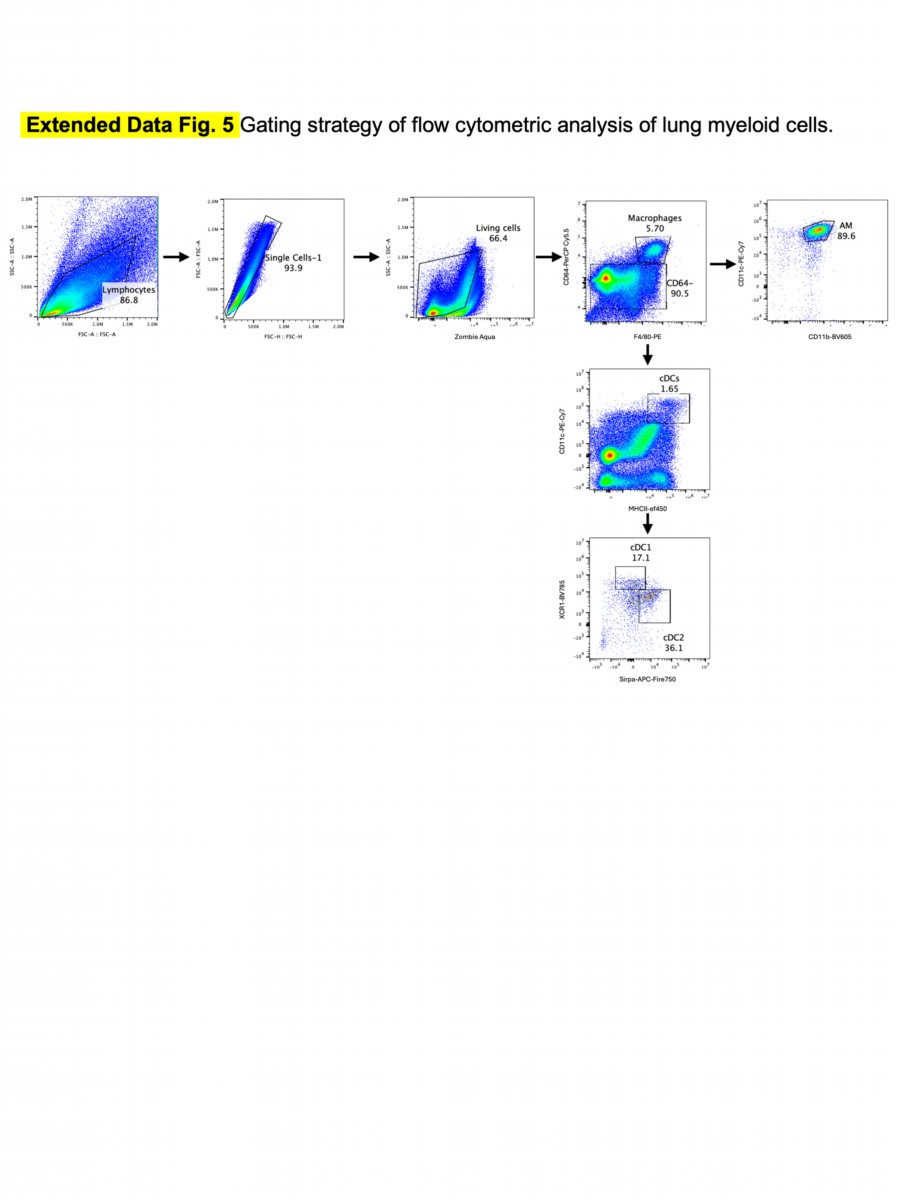


**Supplementary Figure 5 Gating strategy of flow cytometric analysis of lung myeloid cells.** The forward- vs side-scattered-light plot was used to exclude cell debris and gate lymphocyte population (1^st^ diagram). Single cells were selected from lymphocyte population by plotting height vs area of forward-scattered-light (FSC-H vs FSC-A) and gating on the diagonal population (2^nd^ diagram). Alive cells from single cell population were defined by exclusion of the fixable viability dye Zombie Aqua-positive cells (3^rd^ diagram). Total macrophages and CD64- cells were selected based on CD64 and F4/80 staining. This population was further refined based on CD11c positivity, and low expression of CD11b. Dendritic cells were selected from the CD64- live cell population based on high expression of CD11c and MHC-II. From this population cDC1 cells were selected based on XCR1 expression and cDC2 cells based on Sirp-α.

1. **Reference**

1. Busch DH, Pamer EG. MHC class I/peptide stability: implications for immunodominance, in vitro proliferation, and diversity of responding CTL. The Journal of Immunology. 1998;160(9):4441-8.

2. Effenberger M, Stengl A, Schober K, Gerget M, Kampick M, Müller TR, et al. FLEXamers: a double tag for universal generation of versatile peptide-MHC multimers. The Journal of Immunology. 2019;202(7):2164-71.

3. Knabel M, Franz TJ, Schiemann M, Wulf A, Villmow B, Schmidt B, et al. Reversible MHC multimer staining for functional isolation of T-cell populations and effective adoptive transfer. Nature Publishing Group US New York; 2002.
